# An Ultrasensitive Voltammetric Genosensor for the Detection of Bacteria *Vibrio cholerae* in Vegetable and Environmental Water Samples

**DOI:** 10.3390/bios13060616

**Published:** 2023-06-04

**Authors:** Dedi Futra, Ling Ling Tan, Su Yin Lee, Benchaporn Lertanantawong, Lee Yook Heng

**Affiliations:** 1 Department of Chemical Sciences, Faculty of Science and Technology, Universiti Kebangsaan Malaysia, Bangi 43600, Malaysia; dedifutra@lecturer.unri.ac.id; 2 Department of Chemistry Education, Faculty of Education, Universitas Riau, Kampus Binawidya Km 12.5, Pekanbaru 28131, Indonesia; 3 Southeast Asia Disaster Prevention Research Initiative (SEADPRI), Institute for Environment and Development (LESTARI), Universiti Kebangsaan Malaysia, Bangi 43600, Malaysia; lingling@ukm.edu.my; 4 Faculty of Applied Sciences, AIMST University, Semeling 08100, Malaysia; su_yin@aimst.edu.my; 5 Biosensors Laboratory, Department of Biomedical Engineering, Faculty of Engineering, Mahidol University, Nakhon Pathom 73170, Thailand; benchaporn.ler@mahidol.edu

**Keywords:** DNA biosensor, gold nanoparticles, sandwich hybridization, silica nanospheres, *Vibrio cholerae*

## Abstract

In view of the presence of pathogenic *Vibrio cholerae* (*V. cholerae*) bacteria in environmental waters, including drinking water, which may pose a potential health risk to humans, an ultrasensitive electrochemical DNA biosensor for rapid detection of *V. cholera*e DNA in the environmental sample was developed. Silica nanospheres were functionalized with 3-aminopropyltriethoxysilane (APTS) for effective immobilization of the capture probe, and gold nanoparticles were used for acceleration of electron transfer to the electrode surface. The aminated capture probe was immobilized onto the Si-Au nanocomposite-modified carbon screen printed electrode (Si-Au-SPE) via an imine covalent bond with glutaraldehyde (GA), which served as the bifunctional cross-linking agent. The targeted DNA sequence of *V. cholerae* was monitored via a sandwich DNA hybridization strategy with a pair of DNA probes, which included the capture probe and reporter probe that flanked the complementary DNA (cDNA), and evaluated by differential pulse voltammetry (DPV) in the presence of an anthraquninone redox label. Under optimum sandwich hybridization conditions, the voltammetric genosensor could detect the targeted *V. cholerae* gene from 1.0 × 10^−17^–1.0 × 10^−7^ M cDNA with a limit of detection (LOD) of 1.25 × 10^−18^ M (i.e., 1.1513 × 10^−13^ µg/µL) and long-term stability of the DNA biosensor up to 55 days. The electrochemical DNA biosensor was capable of giving a reproducible DPV signal with a relative standard deviation (RSD) of <5.0% (*n* = 5). Satisfactory recoveries of *V. cholerae* cDNA concentration from different bacterial strains, river water, and cabbage samples were obtained between 96.5% and 101.6% with the proposed DNA sandwich biosensing procedure. The *V. cholerae* DNA concentrations determined by the sandwich-type electrochemical genosensor in the environmental samples were correlated to the number of bacterial colonies obtained from standard microbiological procedures (bacterial colony count reference method).

## 1. Introduction

Water is an essential element for human activities such as industrial production, agriculture, recreation, navigation, hydroelectric power generation, and irrigation. It is also a very important medium for the survival of living organisms like fish, bacteria, plankton, etc. Rapid population growth and urbanization, however, contributed to polluting surface and ground waters and caused hundreds of millions of people to lack access to safe drinking water [1]. Contaminated water may act as a source for disease transmission and lead to the outbreak of water-related diseases, such as the cholera parasitic disease and the Hepatitis E viral infection [2,3].

Cholera, a waterborne disease, is an acute intestinal infection marked by exhaustive diarrhea due to ingestion of foods or waters contaminated with the pathogenic *Vibrio cholerae* (*V. cholerae*) bacteria. Cholera infection can be endemic, epidemic, or pandemic, especially after a natural disaster, such as a flood, or in areas with poor sanitation. It produces enterotoxins responsible for watery diarrhea. It is non-virulent, and only serogroups O1 and O139 strains contribute to the widespread epidemic of cholera [4,5]. The first outbreak of cholera was reported by John Snow in 1854, when the Thames river water was polluted with raw sewage containing *V. cholerae* bacteria, causing a cholera epidemic in London [6]. The largest number of cholera cases were reported in Africa in 1996, with 6216 deaths recorded [7]. In connection with this, regular determination and identification of *V. cholerae* in environmental waters become crucial to preventing cholera infection in humans.

Polymerase chain reaction (PCR) [8,9] and microarray techniques [10] are common standard methods used for the specific detection of *V. cholerae* DNA. However, their main weaknesses are that they are time-consuming and involve using multiple and toxic chemicals that can possibly inhibit or interfere with the PCR amplification process. Therefore, specific detection of pathogenic cholera infectious agents caused by *V. cholerae* bacteria with a fast and simple assay system is highly demanded. In terms of simplicity, an electrochemical DNA biosensor would be a good candidate as a substitute tool for these sophisticated conventional methods by virtue of its high sensitivity, fast kinetics, easy-to-handle nature, and low cost [11].

Various nanomaterials have been used in conjunction with DNA biosensors to enhance their sensitivity towards the specific genes ascribed to the biocompatibility and large binding surface area of the nanomaterials. Silica nanospheres, in particular, have received great interest for the construction of biosensors based on the electrochemical strategy for ultrasensitive monitoring of DNA due to their high stability, rich surface chemistry, and facile preparation. Bae et al. [12] have been employing tris(bipyridine)ruthenium(II) [Ru(bpy)_3_^2+^]-doped silica nanospheres for the detection of DNA hybridization based on a thiolated DNA probe-modified gold electrode and demonstrated a wide linear response range from 10 fM to 10 pM. In a separate study, Chang et al. [13] have also made use of the Ru(bpy)_3_^2+^-doped silica nanoparticles to label the DNA probe immobilized on the polypyrole (Ppy)-modified platinum electrode, and the DNA hybridization event was investigated via an electrogenerated chemiluminescence strategy. A low detection limit at the pM level was achieved. Cha et al. [14] developed a microcantilever DNA biosensor based on rhodamine B isothiocyanate-modified silica nanoparticles for the detection of Hepatitis B virus (HBV) DNA, and a lower detection limit was obtained at <3 fM with good selectivity. A monolayer of silica nanoparticles on the cysteine-modified gold electrode was successfully constructed by Zhang et al. [15] for sequence-specific detection of calf thymus DNA using tris(1,10-phenanthroline)cobalt(III) [Co(phen)_3_^3+^] DNA hybridization indicator. A low detection limit was acquired at the pM level. The improvements in the DNA biosensor performance with respect to limit of detection (LOD), dynamic linear range, and selectivity, as shown by the application of silica nanoparticles as immobilization carriers, affirmed the unique structural features of silica nanoparticles for efficient binding of capture probes at high loading capacities.

In this study, we have fabricated an electrochemical DNA biosensor based on silica-gold (Si-Au) nanocomposite-modified screen-printed carbon electrode (SPE) for rapid and specific detection of *V. cholerae* DNA through a sandwich DNA detection process. Silica nanoparticles were derivatized with amine functional groups using 3-aminopropyltriethoxysilane (APTS) via silanization reaction for subsequent cross-linking with capture probes that use glutaraldehyde (GA). The gold nanoparticles were incorporated to enhance the electron transfer rate at the electrode-electrolyte interface. A reporter probe was introduced following the target DNA to the immobilized capture probe in order to amplify the electrochemical signal in the presence of the electroactive anthraquinone oligonucleotide label. Optimization of the Si-Au nanocomposite-based DNA biosensor was performed with differential pulse voltammetry (DPV) for electrochemical voltametric *V. cholerae* DNA detection.

## 2. Materials and Methods

### 2.1. Apparatus and Electrodes

All the electrochemical measurements were realized using the Autolab PGSTAT 12 potentiostat (Metrohm, Herisau, Switzerland). Differential pulse voltammetry (DPV) was carried out in the potential range of −0.85 V to −0.15 V with a step potential of 0.02 V. The three-electrode system consists of a carbon screen-printed electrode (SPE, Scrint Technology (M) Sdn. Bhd., Sungai Petani, Malaysia) modified with Si-Au nanocomposite and DNA as a working electrode, a rod-shaped glassy carbon auxiliary electrode, and an Ag/AgCl reference electrode. All the measured potentials were reported for the Ag/AgCl electrode at room temperature (25 °C), and the chemical solution was homogenized with the Elma S30H sonicator bath. The size of the as-synthesized silica nanospheres was evaluated using a scanning electron microscope (SEM, LEO 1450VP).

### 2.2. Chemicals

All the chemical reagents were of analytical grade and used as received without further purification. Stock solutions of 100 µM capture and reporter probes (Sigma, Singapore) were diluted with 0.05 M potassium phosphate (K-phosphate) buffer at pH 7.0, while a 100 µM target DNA stock solution (Sigma) was diluted with 0.05 M sodium phosphate (Na-phosphate) buffer at pH 7.0. Non-complementary DNA (ncDNA, Sigma) and mismatch DNA (Sigma) solutions were also diluted using 0.05 M Na-phosphate buffer at pH 7.0. The base sequences of oligonucleotides utilized in the present study are summarized in Table 1. The sequences of both capture and reporter probes and the complementary target used in this study were designed based on the *lol*B gene of *V. cholerae* (GenBank accession number AF227752.1), which has been published in our previous research elsewhere [16]. 0.05 M K-phosphate buffer (pH 7.0) was prepared by mixing 0.1 M dipotassium phosphate (K_2_HPO_4_, Fluka) with 0.1 M potassium dihydrogen phosphate (KH_2_PO_4_, Fluka) in deionized water and adjusting to the required pH value. Glutaraldehyde (GA, 25%), 3-aminopropyltriethoxysilane (APTS, 99%), gold nanoparticle powder (<100 nm), and sodium chloride (NaCl) were supplied by Sigma-Aldrich. Ammonia solution (25%), ethanol, and sodium hydroxide (NaOH) were purchased from Systerm. Anthraqinone-2-sulfonic acid monohydrate sodium salt (AQMS) and tetraethoxysilane (TEOS) were obtained from Acrosss and Fluka, respectively.

### 2.3. Synthesis of Silica Nanospheres

Silica nanoparticles were synthesized via the emulsification technique, according to the method reported by Zhang et al. [15] and Niculescu [17] with slight modifications. Briefly, a mixture consisting of deionized water (2 mL) and ammonia solution (5 mL) was sonicated for 10 min at 25 °C. About 2 mL of TEOS and 4 mL of ethanol were then added and sonicated for another 30 min at 55 °C. Following that, around 2 mL of APTS was added to the mixture, and it was kept stirring overnight at ambient conditions. The amine-functionalized silica nanoparticles were finally collected by centrifugation at 4000 rpm for 30 min and thoroughly washed with ethanol and K-phosphate buffer (0.05 M, pH 7.0) three times before being air-dried at room temperature. Approximately 7 mg of dried APTS-modified silica nanospheres were then dispersed in 500 μL of ethanol for further usage.

### 2.4. Fabrication of DNA Biosensor Based on Si-Au Nanocomposite Electrode

**Figure 1:**
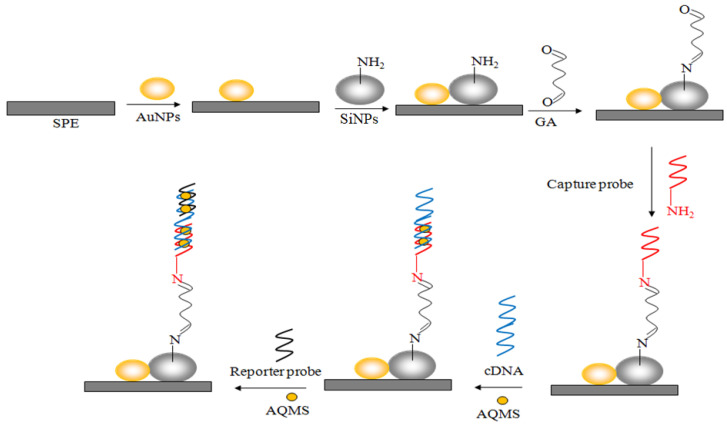
Schematic illustration of the electrochemical genosensor fabrication process based on Si-Au nanocomposite-modified carbon SPE for voltammetric sandwich DNA detection of toxigenic bacteria, *V. cholerae* DNA sequences.

The fabrication of the *V. cholerae* DNA biosensor was carried out by first depositing 10 µL of gold nanoparticle (1 mg/300 µL) suspension in ethanol onto a carbon SPE and leaving it to dry at room temperature. Following that, about 2 µL of silica nanosphere suspension was deposited onto the gold nanoparticle-modified SPE and air-dried at 25 °C. The resulting Si-Au nanocomposite-modified SPE (Si-Au-SPE) was then dipped in 400 µL of 5% glutaric dialdehyde for an hour to activate the amine groups on the silica nanoparticles’ surfaces and washed with plentiful amounts of K-phosphate buffer (0.05 M, pH 7.0). The Si-Au-SPE was later immersed in 300 µL of 2 µM capture probe solution at 2 °C for 5 h to permit cross-linking of DNA capture probe with amine-functionalized silica nanospheres. Following that, the electrode was washed with abundant K-phosphate buffer (0.05 M, pH 7.0) to remove the unbound capture probe. Next, the immobilized capture probe was soaked in 300 µL of complementary DNA (cDNA) solution containing 1 M NaCl and 1 mM AQMS for 45 min to allow the DNA hybridization reaction to take place and rinsed thoroughly with 0.1% SDS several times. The double-stranded DNA (dsDNA)-immobilized Si-Au-SPE was then incubated in 300 µL of 2 µM reporter probe solution containing 1 M NaCl and 1 mM AQMS for another 45 min, followed by washing with copious amounts of 0.05 M Na-phosphate buffer (pH 7.0), and immersed in 400 µL of 0.1% SDS for 5 min to remove excess reporter probe sequence before DPV measurement. A DNA biosensor without an immobilized reporter probe was also prepared in the same manner for comparison purposes. The stepwise voltammetric *V. cholerae* genosensor fabrication process is illustrated in Figure 1. All the DPV measurements were performed in 4.5 mL of K-phosphate buffer (0.05 M, pH 7.0) at 25 °C.

### 2.5. Optimization of Voltammetric V. cholerae Genosensor Response

The DNA biosensor response was optimized with a spectrum of parameters in order to obtain the best working conditions for sequence-specific quantification of *V. cholerae* DNA. The loading effects of both gold and silica nanoparticles were optimized from 0.001–0.010 mg and 0.002–0.020 mg, respectively, on the carbon SPE. The capture probe was loaded between 0.1 µM and 4.0 µM on the Si-Au-SPE, while the reporter probe was varied between 0.01 µM and 3.00 µM. The capture probe immobilization time was optimized with the capture probe-immobilized Si-Au-SPE being dipped in a 2 µM capture probe solution from 1 h to 16 h.

Optimization of the sandwich DNA hybridization time, on the other hand, was investigated by immersing the DNA biosensor into 1 × 10^−6^ µM cDNA solution for 5–240 min and subsequently soaking it in the 2 µM reporter probe solution for 5–240 min. The ionic effect on the DNA hybridization reaction was examined by varying the NaCl concentration in the DNA hybridization medium (i.e., 0.05 M Na-phosphate buffer at pH 7.0) between 0.1 M and 2.5 M. The optimum pH and buffer capacity were determined by changing the pH and concentration of Na-phosphate buffer from pH 5.5–8.5 and 0.01–0.30 M, respectively.

### 2.6. Sandwich DNA Hybridization Detection

The *V. cholerae* DNA biosensor response was monitored at a series of target DNA concentrations from 1.0 × 10^−19^ to 1.0 × 10^−5^ M at pH 6.5 in order to obtain the linear response range and the lowest detection limit of the DNA biosensor. The DPV response was recorded after 45 min of the DNA hybridization reaction at 25 °C. Reproducibility of the DNA biosensor was assessed by batch-producing ten replicate DNA biosensors using the same batch of precursor chemicals in a similar preparation manner under the same experimental conditions and testing them with two different cDNA concentrations, i.e., 1.0 × 10^−11^ and 1.0 × 10^−9^ M.

For the selectivity study, the *V. cholerae* DNA biosensor was exposed to three bases of mismatched DNA, ncDNA, and cDNA at concentrations of 4.0 × 10^−6^ M and 4.0 × 10^−7^ M, respectively. The stability of the DNA biosensor based on Si-Au nanocomposite electrodes was determined by batch-fabricating some 52 units of DNA electrodes and storing them in a refrigerator at 4 °C. The voltammetric signals of three units of DNA biosensors towards the detection of 1.0 × 10^−10^ M of cDNA were periodically taken within 90 days of the experimental period. Regeneration of the *V. cholerae* DNA biosensor was conducted by incubating the DNA electrode in 1.0 × 10^−6^ M cDNA for 30 min at room temperature, and the DPV signal was measured thereafter. The DNA biosensor was then soaked in 0.1 M NaOH regeneration solution for 15 min and washed thoroughly with 0.05 M K-phosphate buffer (pH 7.0) for 60 s before the next DPV measurement was carried out. DNA rehybridization with 1.0 × 10^−6^ M cDNA was later performed again for 30 min at 25 °C. The same experimental procedures were repeated five times for DNA rehybridization and regeneration of the *V. cholerae* DNA biosensor.

### 2.7. Determination of V. cholerae DNA in Various Bacterial Strains, Environmental Water, and Vegetable Samples

Different strains of *V. cholerae* bacteria (e.g., *J3324*, *J2126*, *KM5802*, *J3330*, *CDHI 5294*, and *UVC1324*) and some other species of bacteria (e.g., *Citrobacter freundii, Enterobacter aerogenes*, and *Klebsiella pneumonia*) were supplied from the Microbiology Laboratory, Faculty of Applied Sciences, AIMST University, Kedah. Genomic DNA extraction was conducted on these bacteria using QIAGEN Genomic-tip 500/G according to the manufacturer’s protocol. The concentration of the extracted DNA was measured using a bio-spectrophotometer (Perkin Elmer 25 UV/VIS). Recovery of *V. cholerae* DNA in the media containing various bacterial strains was carried out by using diluted *V. cholerae* cDNA in the concentration range of 8.50 × 10^−4^ to 6.50 × 10^−9^ µg/µL in Na-phosphate buffer (0.05 M, pH 6.5).

Meanwhile, about five river water samples were directly collected from the Langat River in the state of Selangor after heavy rain. Approximately 1 mL of each river water sample was sonicated for 20 min at 25 °C to release the *V. cholerae* DNA and immediately measured with the fabricated DNA biosensor without dilution. In addition, about five cabbage (*Brassica oleracea* L.) samples were obtained from the domestic market. One gram of each vegetable sample was then immersed in 10 mL of sterile deionized water followed by a vortex at room temperature for 5 min; then the solution was isolated for direct determination of *V. cholerae* DNA with the developed electrochemical DNA biosensor.

Determination of *V. cholerae* bacteria in river water and cabbage samples was also carried out by using alkaline peptone water (APW) as an enrichment broth and thiosulfate citrate bile salts sucrose (TCBS) agar as the selective agar medium for the isolation of *V. cholerae* bacterial colonies. For cDNA concentration recovery with the developed DNA biosensor, both river water and vegetable samples were spiked with 9.21 × 10^−6^ µg/µL *V. cholerae* target DNA and evaluated with the proposed DNA biosensor. All the experiments were carried out in triplicate at room temperature.

## 3. Results and Discussion

### 3.1. Silica Nanospheres and Sandwich-Type Voltammetric DNA Biosensor Characteristic Response

**Figure 2:**
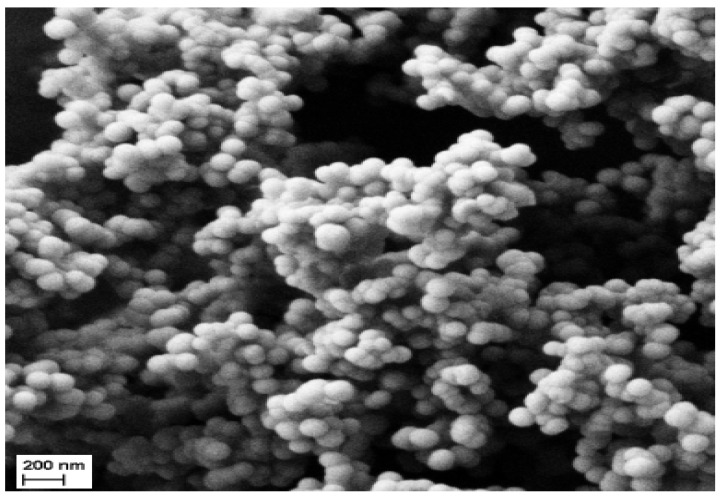
The morphology of the silica nanospheres synthesized via the emulsification technique was captured under scanning electron microscopy.

The silica nanospheres were synthesized by using a standard water-in-oil nanoemulsification method based on a self-assembly system. In the presence of ethanol and ammonia, the silica particles were produced via hydrolysis and condensation reactions and supported by the sonication technique to create the nano-sized spheres. The surface of silica nanospheres was functionalized with an amine group through the silanization reaction simply by adding APTS to the silica precursors. The size and morphology of the silica nanoparticles were examined with SEM (Figure 2). The stable aqueous suspensions of organically modified silica nanospheres have an average size smaller than 100 nm. No aggregation of APTS-modified silica nanospheres in the aqueous solution was observed. The highly monodispersed silica nanospheres were attributed to the electrostatic force held between the nanoparticles. The uniformity of the silica nanospheres is crucial to the good stability, sensitivity, and reproducibility of the electrochemical DNA biosensor.

The characterization of the silica nanoparticles had been reported before [18,19]. Chemical elucidation of the amine-functionalized silica nanospheres by Fourier transform infrared spectroscopy (FTIR) identified chemical bonds, such as N-H (amine) bending vibration at 1635 cm*^−^*^1^; the aliphatic Si-O functional group of the APTS-modified nanosilica at the absorption bands of 1047 cm*^−^*^1^ and 957 cm*^−^*^1^; and the stretching bands of aliphatic –OH and Si-C were observed at the characteristic peaks at the wave numbers of 3379 cm*^−^*^1^ and 793 cm*^−^*^1^, respectively [18]. X-ray diffraction (XRD) analysis of the as-synthesized APTS-modified silica nanoparticles revealed clear and broad spectral peaks at 2θ around 13° and 23°, which suggests that the silica nanoparticles were in the amorphous form [19].

**Figure 3:**
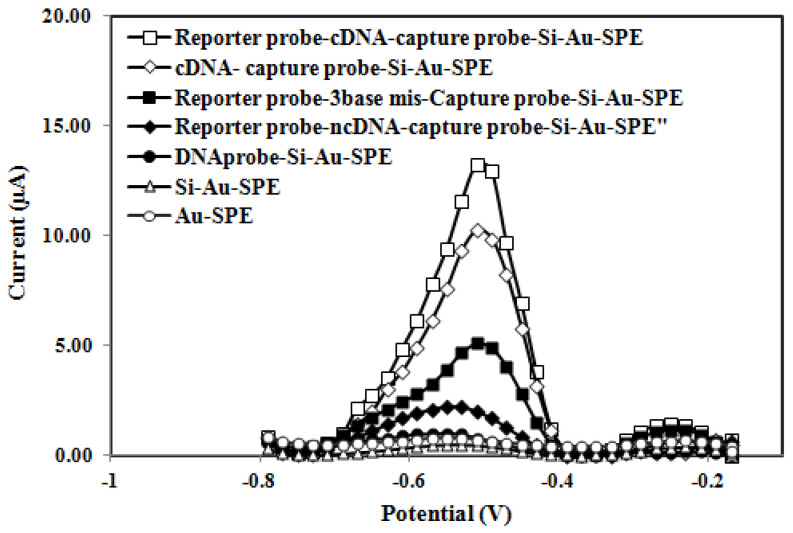
Differential pulse voltammograms of the AQMS redox indicator on the gold nanoparticle−, Si-Au nanocomposite−, and DNA−modified carbon SPE. The DPV measurement was carried out in 0.05 M K-phosphate buffer (pH 7.0) at a scan rate of 0.5 Vs*^−^*^1^ versus the Ag/AgCl reference electrode.

The monitoring principle of the voltammetric genosensor is based on the current produced by the hybridization of the probe and target DNA and the intercalation of AQMS. A time-dependent potential is applied to the electrochemical cell consisting of the DNA, counter, and reference electrodes. It measures the resulting current from the redox response of the AQMS as a function of that potential, thus providing quantitative information about the *V. cholerae* cDNA concentration. The DPV peak current signal of the *V. cholerae* DNA biosensor based on Si-Au nanocomposite SPE, which was indicated by the AQMS redox label before and after DNA sandwich hybridization with cDNA, ncDNA, and 3-base mismatched DNA, is shown in Figure 3. The highest DPV peak current signal was obtained for the reporter probe hybridized with the immobilized dsDNA that consisted of the immobilized DNA probe and cDNA on the Si-Au-SPE due to the complete sandwich DNA hybridization reaction followed by full AQMS intercalation. This signifies that the capture probe has been successfully grafted onto the silica nanospheres via covalent bonding. In the absence of the reporter probe, the dsDNA-immobilized Si-Au-SPE gave a fairly high DPV current response but was somewhat lower than that of the sandwich DNA hybridized electrode as the full DNA hybridization reaction was not accomplished. The 3-base mismatched DNA-modified Si-Au-SPE demonstrated a noticeable DPV current signal as it possessed 90% bases complementary to both reporter and capture probes. A fairly low DPV peak current response was seen in the ncDNA-modified Si-Au nanocomposite electrode as the ncDNA strand was not fully complementary, thereby failing AQMS intercalation into dsDNA. The capture probe-immobilized Si-Au-SPE gave negligible DPV response due to the electrostatic repulsion that occurred between the negatively charged AQMS and the immobilized capture probe. Negligible DPV responses were also obtained for both Si-Au-SPE and Au-SPE, which imply no non-specific adsorption of the AQMS DNA hybridization label on the electrode surface. The positively-charged anthraquinone DNA hybridization indicator was used as a redox probe for the electrochemical transduction of DNA hybridization due to the fact that cations could intercalate more rapidly than anions and neutral species (such as ferrocene, a neutral organometallic compound) to the negatively charged DNA backbone, which would enhance the possibility of non-specific signals [20].

### 3.2. Optimization of Electrochemical DNA Biosensor Response for Toxigenic Bacterium V. cholerae Detection

**Figure 4:**
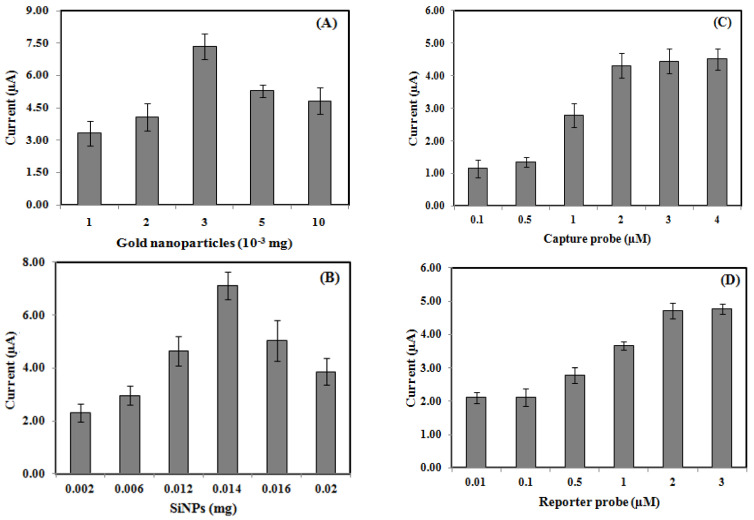
Effects of gold nanoparticles (**A**), silica nanospheres (**B**), capture probes (**C**), and reporter probe loadings (**D)** on the electrochemical sandwich-type *V. cholerae* DNA biosensor DPV response using a 1 mM AQMS DNA hybridization label.

The effects of gold nanoparticles, silica nanospheres, the capture probe, and reporter probe loadings on the voltammetric DNA biosensor response are presented in Figure 4. As silica nanoparticles are essentially non-conducting, they would block the electron transfer from the DNA hybridization redox indicator to the electrode surface. As such, gold nanoparticles were coated onto the carbon SPE surface to facilitate the electron transfer reaction. The DNA biosensor response increased as the gold nanoparticle loading increased from 0.001 mg to 0.003 mg (Figure 4A) due to the increased electron transfer rate from the AQMS redox label to the Si-Au-SPE surface. Upon loading the amount of gold nanoparticles above 0.003 mg, the excess coverage of the gold nanoparticles on the SPE active surface has made the electron transfer rather limited, and the DNA biosensor response has become lower. For the silica nanosphere loading effect, the DNA biosensor signal increased gradually with the increasing silica nanosphere loading from 0.002–0.014 mg (Figure 4B), which allowed a higher number of capture probes attached to the silica nanospheres’ surfaces for subsequent DNA sandwich hybridization with cDNA and reporter probe and intercalation with AQMS at a higher reaction rate. As the amount of silica nanospheres increased from 0.014 to 0.020 mg, the DNA biosensor response declined as a result of sluggish electron transfer between insulative silica nanoparticles. The optimized Si-Au-SPE was then utilized for further optimizing the capture probe and reporter probe loadings. Figure 4C,D indicated that the DPV response of the DNA biosensor achieved a saturation state when 2.0 µM of both capture and reporter probes were introduced. The steady-state voltammetric response attained by the electrochemical genosensor suggested that the silica nanospheres’ surfaces have been immobilized with the capture probe (equivalent to the number of ssDNA molecules of 1.7885 × 10^12^). For optimum DNA sandwich hybridization with target DNA and a signal probe, 2.0 µM capture and reporter probes were employed in the following DNA biosensor optimization studies.

**Figure 5:**
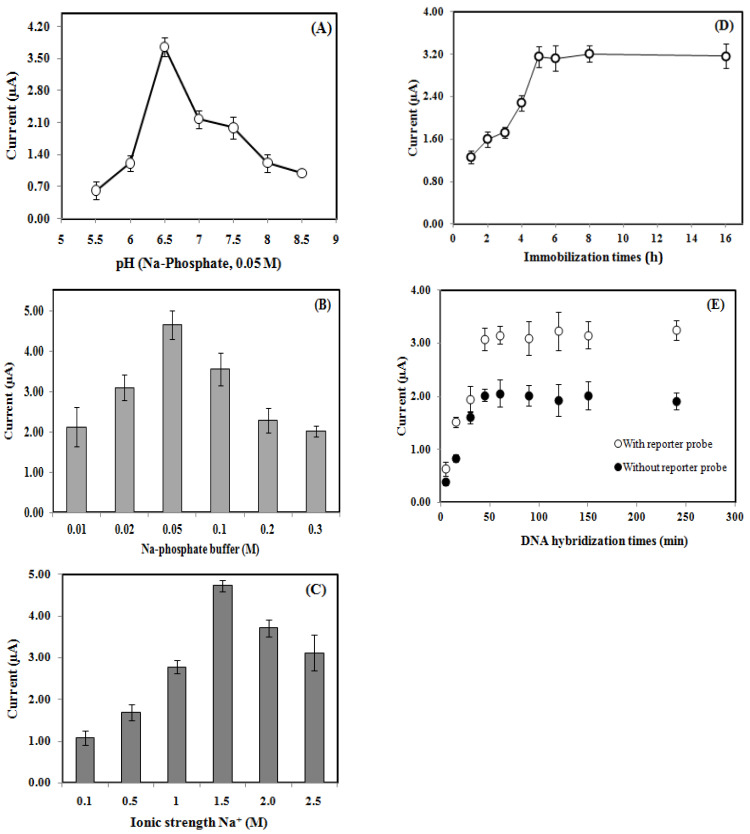
The pH (**A**), buffer capacity (**B**), ionic strength (**C**), capture probe immobilization time (**D**), and DNA hybridization duration (**E**) profiles of the voltammetric genosensor based on Si-Au-SPE towards sandwich DNA detection of *V. cholerae*.

The pH, buffer capacity, and ionic strength of the DNA hybridization medium are crucial for an optimum DNA sandwich hybridization reaction. As can be seen in Figure 5A, the DPV peak current signal of the DNA biosensor increased from pH 5.5 to pH 6.5 as a mildly acidic DNA hybridization buffer could promote maximum DNA hybridization reaction. In contrast, a highly acidic pH level would result in the protonation of the DNA phosphodiester bond and reduce the DNA molecule’s solubility [21]. Consequently, it interferes with the DNA sandwich hybridization reaction rate. Whereas in alkaline conditions, the DNA biosensor response progressively decreased. This was due to the fact that the deprotonation of the DNA sugar-phosphate backbone has caused an increasing electrostatic repulsion between negatively charged DNA molecules. In general, a highly acidic or basic medium could result in denaturation of dsDNA [22]. Therefore, Na-phosphate buffer at pH 6.5 was chosen as the optimum pH for the DNA sandwich hybridization reaction.

Appropriate buffer capacity and ionic strength could retain the pH and charge balance of the solution to yield maximum hybridization of DNA. Na-phosphate buffer containing Na^+^ ions appeared to be the most suitable DNA hybridization buffer and gave the best ionic strength effect compared to the use of K-phosphate or Tris-HCl buffer containing K^+^ or Mg^2+^ ions, respectively. The DNA biosensor yielded maximal response when 0.05 M Na-phosphate buffer (Figure 5B) and 1.5 M Na^+^ ions were used in the DNA sandwich hybridization reaction (Figure 5C). The Na^+^ ions interacted with the negatively charged backbone phosphate groups of DNA and neutralized the electrostatic repulsion between DNA molecules [23,24], thereby facilitating the hybridization reaction with the cDNA strand. Further increases in the Na-phosphate buffer and Na^+^ ion concentrations above 0.05 M and 1.5 M, respectively, reduced the DNA biosensor response owing to the superabundance of salt content, which discouraged the DNA sandwich hybridization process.

For the capture probe immobilization duration study, the DNA biosensor response was observed to slowly increase with capture probe immobilization duration from 1.0 h to 5.0 h and stabilize thereafter until the 16.0 h capture probe immobilization period (Figure 5D). The longer the DNA immobilization period, the higher the number of capture probes immobilized onto the Si-Au-SPE surface.

The steady-state response achieved from 5.0 h of immobilization duration and onwards implies that the Si-Au nanocomposite electrode surface has been entirely immobilized with the capture probes, and further prolonging the immobilization duration would no longer increase the DNA biosensor response. Hence, a 5.0-hour immobilization time was selected for optimum capture probe immobilization on the Si-Au-SPE. For the DNA sandwich hybridization time determination study, the DNA biosensor responded to cDNA in the presence and absence of the reporter probe. The DNA biosensors that responded to both cDNA and reporter probes showed a higher DPV current response as they involved complete DNA sandwich hybridization and AQMS intercalation reactions. However, the DNA biosensor, whether or not it responded to the reporter probe, showed an optimum DNA hybridization period of 45 min (Figure 5E), which indicates that the immobilized capture and reporter probes have been fully hybridized with cDNA.

### 3.3. Dynamic Linear Response of the Voltammetric Genosensor towards Sandwich DNA Detection of Toxin-Producing Bacteria V. cholerae

**Figure 6:**
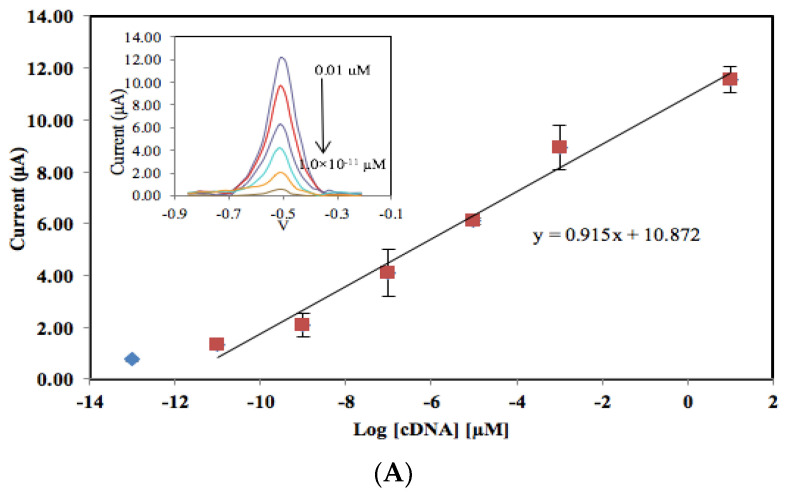

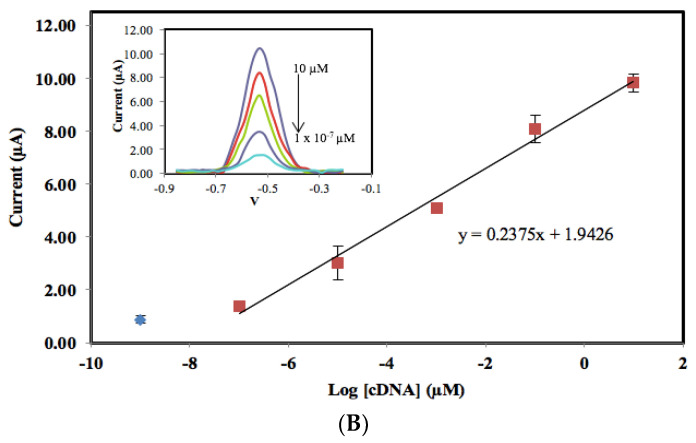
The dynamic linear response range of the *V. cholerae* DNA biosensor at the presence (**A**) and absence of a reporter probe (**B**) using various cDNA concentrations from 1.0 × 10^−5^ to 1.0 × 10^−19^ M with a 45−minute DNA hybridization time at 25 °C.

The dynamic linear concentration range of the *V. cholerae* DNA biosensor was investigated in the presence and absence of the reporter probe with various cDNA concentrations ranging from 10 to 1.0 × 10*^−^*^13^ µM (i.e.*,* 9.21 × 10*^−^*^2^–9.21 × 10*^−^*^16^ µg/µL) (Figure 6). The DNA biosensor response increased proportionally with the increasing cDNA concentration due to the increasing DNA sandwich hybridization reaction and AQMS intercalation on the DNA electrode based on Si-Au-SPE. The reporter probe-modified Si-Au-SPE demonstrated a two orders of magnitude wider linear response range with a 10,000 times lower LOD compared to the one without the reporter probe (Table 2). This might best be explained by the fact that the complete DNA sandwich hybridization process between cDNA and the capture/reporter probe has extended the length of immobilized dsDNA to allow higher loading of intercalated anthraquinone redox label, leading to a higher DPV signal and improvement in the overall *V. cholerae* DNA biosensor sensitivity.

The DNA biosensor constructed based on a Si-Au nano-composite electrode was then applied to evaluate its selectivity towards the detection of 3-base mismatched DNA, ncDNA, and cDNA at 4.0 µM and 0.4 µM (Table 3). About 46.0–52.1% of the DPV peak current response was obtained with the *V. cholerae* DNA biosensor towards the hybridization with 3-base mismatched DNA compared to its response with cDNA. This is because the 3-base mismatched DNA contains 27 bases that could hybridize with the capture and reporter probes. Whereas negligible DPV response is expected for the evaluation of ncDNA with the developed *V. cholerae* sandwich-type electrochemical DNA biosensor.

The *V. cholerae* DNA biosensor based on the Si-Au-SPE exhibited a large improvement in terms of sensitivity, dynamic range response, and detection limit (LOD) as compared to the previously reported amperometric *V. cholerae* DNA biosensor based on a gold electrode [25], a gold nanoparticle-modified glass electrode [26], and gold nanoparticle-modified SPE and magnetic beads [27] for DNA sequence-specific detection and quantification of pathogenic *V. cholerae* microbes (Table 4). The promising electrochemical *V. cholerae* DNA biosensor performance based on Si-Au nanocomposite SPE is chiefly because of the highly monodispersed and homogenous nano-sized silica nanospheres that contributed no diffusion barrier and the extremely large surface area available, which allowed immobilization of a large number of DNA probes.

### 3.4. Regeneration and Stability of the V. cholerae DNA Biosensor Based on Si-Au Nanocomposite SPE

**Figure 7:**
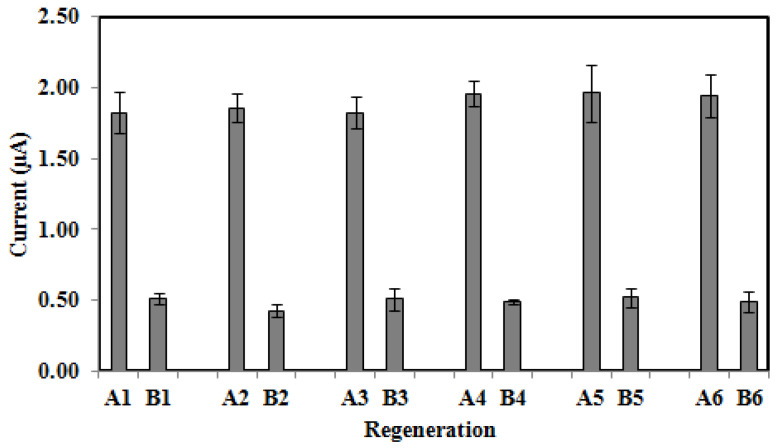
Regeneration behaviour of the *V. cholerae* DNA biosensor using 0.1 M NaOH regeneration solution (15 min exposure). A1 is the DPV response before regeneration and A2-A6 are current responses from rehybridization (in 1.0 × 10^−6^ M cDNA for 30 min).The DPV current of B1–B6 are the responses after regeneration processes without rehybridization to indicate that the process of regeneration has occurred.

The regeneration profile of the DNA biosensor is depicted in Figure 7. The DNA biosensor response from hybridization (A1) was lost after dipping in 0.1 M NaOH for 15 min (B1). This indicated that the hydrogen bonds between the base pairs of the dsDNA helix were ruptured in the 0.1 M NaOH followed by cleavage of the immobilized sandwich hybridized DNA duplexes. When the DNA biosensor was rehybridized with 1.0 × 10^−6^ M cDNA, the DNA biosensor was found capable of retaining its initial response after regeneration (A2). By repeating the regeneration processes another five times, the current responses were similar (A3-A6) demonstrating good reversibility with relative standard deviation (RSD) values in the range of 5.0–7.7% (RSD, *n* = 6). Thus, the biosensor can be regenerated with NaOH for subsequent applications.

**Figure 8:**
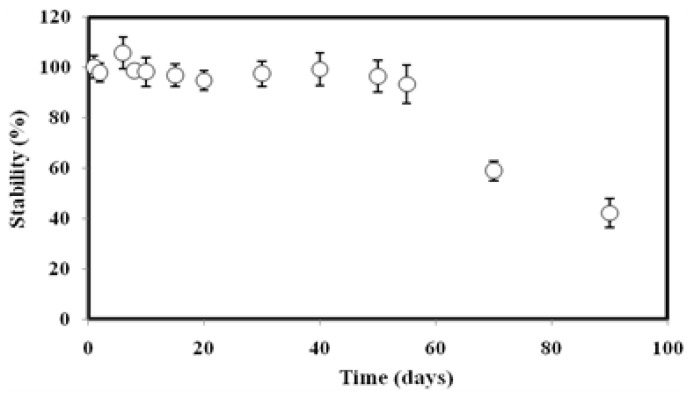
The stability behaviour of the DNA biosensor for the determination of *V. cholerae* DNA over a period of 100 days.

The lifespan of the DNA biosensor based on Si-Au-SPE is illustrated in Figure 8. The DNA biosensor response was stable up to 55 days of storage at 4 °C, with 93.4% of its initial current signal still achievable. The DNA biosensor response was then dropped to 59.0–42.2% between 70 and 90 days of storage time compared to its original current response acquired on the first day. The long lifetime of the Si-Au-SPE-based DNA biosensor has proven the excellent biocompatibility of the silica nanoparticle matrix for maintaining the bioactivity of the immobilized capture probe and allowing efficient transduction of the biorecognition event.

### 3.5. Electrochemical DNA Biosensor for Environmental Applications via Sandwich Hybridization System

To evaluate the feasibility of the proposed DNA biosensor based on Si-Au nanocomposites electrode in determining *V. cholerae* DNA concentration through the sandwich hybridization strategy, various bacterial strains, environmental water samples, and vegetable samples were used for the evaluation of the developed DNA biosensor. The bacterial samples were grown aerobically on the Luria-Bertani medium and extracted to determine the DNA concentrations. The DNA concentrations for the respective *V. cholerae* strains, such as *J3324*, *J2126*, *KM5802*, *J3330*, *CDHI5294,* and *UVC1324,* were 1.60 µg/µL, 0.63 µg/µL, 1.20 µg/µL, 0.71 µg/µL, 0.50 µg/µL, and 1.10 µg/µL, respectively. Meanwhile, the DNA concentrations obtained for the other bacterial species, namely *C. freundii, E. aerogenes*, and *K. pneumonia,* were 0.57 µg/µL, 0.53 µg/µL, and 0.62 µg/µL, respectively.

The concentration of the extracted DNA was then adjusted to 1.0 × 10^−4^ µg/µL DNA by using Na-phosphate buffer (0.05 M, pH 6.5) and measured with the DNA biosensor. Based on the results tabulated in Table 5, the developed DNA biosensor was found to be highly selective towards the detection of *V. cholerae UVC1324* and *V. cholerae J3330*, while exhibiting the lowest DPV response against *E. earogenes, C. feundii,* and *K. pneumoniea* bacteria. The extracted DNA samples for *V. cholerae UVC1324*, *V. cholerea J3330*, *C. freundii, E. aerogenes*, and *K. pneumonia* bacteria were then diluted using the same buffer containing *V. cholerae* cDNA in the concentration range of 8.50 × 10^−4^ to 6.50 × 10^−9^ µg/µL and measured again using the proposed DNA biosensor for recovery of the *V. cholerae* cDNA concentration, and satisfactory recovery values were obtained between 94.0% and 102.0% for *V. cholerae UVC1324* and *V. cholerea J3330* (Table 6).

On the other hand, the presence of *V. cholerae* DNA in river water and cabbage samples was first evaluated with the developed sandwich-type voltammetric DNA biosensor, followed by verification with the bacterial colony growth experiment. Based on the data summarized in Table 7, the *V. cholerae* DNA concentrations were obtained in the range of 1.20 × 10*^−^*^10^ to 4.71 × 10*^−^*^13^ µg/µL for river water samples 2 and 5, and 4.22 × 10*^−^*^13^–9.46 × 10*^−^*^13^ µg/µL for cabbage samples 3 and 5. The bacterial colony growth experiment confirmed the presence of *V. cholerae* bacteria in the range of 12*–*36 colonies in river water samples 2 and 5, and 8–14 colonies in cabbage samples 3 and 5. No detectable bacterial colonies were found in the river water samples 1 and 3, or cabbage samples 1, 2, and 4. For the recovery of *V. cholerae* DNA spiked into the Langat river water and cabbage samples, satisfactory recoveries between 96.5 and 101.6% were obtained (Table 8).

## 4. Conclusions

The determination of *V. cholerae* DNA via sandwiched DNA hybridization was successful and applied to the analysis of some environmental samples. The use of silica nanospheres increased the surface area for the DNA hybridization process and allowed good diffusion of analytes on the Si-Au-SPE electrode surface. Compared with DNA detection without reporter probes, the use of reporter probes for sandwich-type DNA hybridization appeared to further improve the DNA biosensor’s performance. Overall, these factors resulted in a large improvement in the sensitivity, detection limit, and linear response range of the DNA biosensor, particularly the detection limit down to the attomolar level with a sensitivity of 0.362 µA/decade. This signifies that the DNA sandwich hybridization strategy between cDNA and the capture/reporter probe on the silica nanosphere surfaces enhanced the voltammetric signal from the redox intercalator AQMS. Investigation of *V. cholerae* DNA in river water and vegetable samples demonstrated approximately 100% recovery, which implied a reliable and accurate DNA biosensor system. Therefore, the DNA biosensor reported here has a good potential for rapid determination of the toxigenic bacterium *V. cholerae* in environmental samples.

## Figures and Tables

**Table 1:** The base sequences of oligonucleotides used in the present research.

DNA	Base Sequences
Capture probe	5′-TCA TCG ACC TGT AAG (AmC3)
Reporter probe	5′-TTC AGC ACG GTT TGA
cDNA	5′-TCA AAC CGT GCT GAA CTT ACA GGT CGA TGA
ncDNA	5′-CGT GGT TTT ACC ATT TGC AAC AGC
3-base mismatched DNA	5′-TCA AAC CGT GCT GAA CTT GTC GGT CGA TGA

**Table 2:** The analytical performance of the sandwich-type electrochemical *V. cholerae* DNA biosensor in the presence and absence of the reporter probe.

Parameters	DNA Biosensor in the Presence of Reporter Probe	DNA Biosensor in the Absence of Reporter Probe
Sensitivity (µA/decade)	0.915 ± 0.005	0.2375 ± 0.002
R^2^	0.9836	0.9823
Dynamic linear range (M)	1.0 × 10^−17^–1.0 × 10^−7^	1.0 × 10^−13^–1.0 × 10^−5^
Detection limit (M)	1.25 × 10^−18^	2.24 × 10^−14^
Repeatability (*n* = 6, RSD %)	3.5–4.7	3.6–4.9

**Table 3:** Selectivity study of the *V. cholerae* DNA biosensor towards sandwich DNA detection of cDNA, ncDNA, and 3-base mismatched DNA at different concentrations using 1 mM AQMS redox indicator.

DNA	DNA Concentration at 4.0 µM		DNA Concentration at 0.4 µM	
	Peak Current (µA, *n* = 4)	Sensitivity (%)	Peak Current (µA, *n* = 4)	Sensitivity (%)
cDNA	13.933	100.00	12.315	100.01
3-base mismatched DNA	7.050	50.61	5.665	46.01
ncDNA	0.817	5.86	1.238	10.05

**Table 4:** Comparison between the developed sandwich-type DNA biosensor and other previously reported electrochemical DNA biosensors for the determination of toxin-producing *V. cholerae* DNA.

Material and Electrode Modification	Linear Range (M)	LOD (M)	Reproducibility (% RSD)	Hybridization Time (min)	Reference
Si-Au-SPE	1.00 × 10^−17^–1.00 × 10^−7^	1.25 × 10^−18^	3.5–4.7 (*n* = 5)	45	This study
AuNPs-modified carbon SPE and reporter probe-modified magnectic beads	-	3.90 × 10^−9^	-	20	Low et al. [27]
AuNPs-modified glass electrode	1.10 × 10^−5^–2.20 × 10^−4^	1.10 × 10^−5^	-	5	Patel et al. [26]
Gold electrode	-	1.00 × 10^−12^–185.00 × 10^−12^	-	30	Yu et al. [25]

**Table 5:** The DPV peak current response of the electrochemical DNA biosensor towards the detection of various *V. cholerae* strains and other species of bacteria via sandwich hybridization strategy.

No.	Bacteria	DNA (µg/µL)	Current (µA)	Baseline ± SD	*t* Test	Current (%)
1	*Citrobacter freundii*	1.0 × 10^−4^	0.336 ± 0.03	0.450 ± 0.03	32.71	13.2
2	*Enterobacter aerogenes*	1.0 × 10^−4^	0.298 ± 0.05	0.450 ± 0.03	34.52	11.7
3	*Klebsiella pneumoniae*	1.0 × 10^−4^	0.391 ± 0.02	0.450 ± 0.03	8.429	15.4
4	*Vibrio cholerae J3330*	1.0 × 10^−4^	2.301 ± 0.14	0.450 ± 0.03	82.24	90.3
5	*Vibrio cholerae CDHI5294*	1.0 × 10^−4^	1.588 ± 0.03	0.450 ± 0.03	27.34	62.4
6	*Vibrio cholerae J2126*	1.0 × 10^−4^	1.388 ± 0.09	0.450 ± 0.03	11.58	54.5
7	*Vibrio cholerae UVC1324*	1.0 × 10^−4^	2.548 ± 0.09	0.450 ± 0.03	84.62	100.0
8	*Vibrio cholerae KMS3802*	1.0 × 10^−4^	1.212 ± 0.04	0.450 ± 0.03	12.63	47.6
9	*Vibrio cholerae J3324*	1.0 × 10^−4^	1.517 ± 0.08	0.450 ± 0.03	26.62	59.5

Note: The critical value, *t*_4_ = 2.78 (*p* = 0.05, 95%).

**Table 6:** Recoveries of *V. cholerae* DNA from various bacterial strains’ media by using the proposed voltammetric genosensor based on Si-Au nanocomposite electrode via sandwich hybridization strategy (*n* = 4).

No.	Bacteria Samples	Real DNA Concentration (µg/µL)	Found (µg/µL)	Recovery (%)
1	Complementary DNA	9.21 × 10^−6^	9.23 × 10^−6^	100.2
2	*Citrobacter freundii*	8.50 × 10^−4^	1.18 × 10^−10^	0.0
3	*Enterobacter aerogenes*	6.50 × 10^−4^	4.96 × 10^−3^	0.2
4	*Klebsiella pneumoniae*	4.50 × 10^−4^	7.41 × 10^−6^	1.7
5	*Vibrio cholerae UVC1324*	5.50 × 10−5	5.43 × 10−5	98.8
6	*Vibrio cholerae UVC1324*	5.50 × 10−6	5.60 × 10−6	101.8
7	*Vibrio cholerae UVC1324*	5.50 × 10−7	5.47 × 10−6	99.5
8	*Vibrio cholerae UVC1324*	5.50 × 10−8	5.43 × 10−8	98.8
9	*Vibrio cholerae J3330*	5.50 × 10−5	5.22 × 10−5	95.0
10	*Vibrio cholerae J3330*	6.50 × 10−9	6.27 × 10−9	96.4

Note: The DNA biosensor linear equation, i.e., y = 0.376x + 5.6058, was used to calculate the *V. cholerae* DNA concentration.

**Table 7:** Determination of *V. cholerae* DNA concentration in Langat river water and cabbage samples by using the sandwich-type electrochemical DNA biosensor and verification with the bacterial colony growth experiment (*n* = 4).

Sample	Current (µA, *n* = 4)	*V. cholerae* DNA Concentration Found (µg/µL)	*V cholerea* (Colony/mL)
River water			
Control	0.453	ND	ND
Sample 1	0.593	ND	ND
Sample 2	1.876	1.20 × 10^−10^	36
Sample 3	0.461	ND	ND
Sample 4	0.971	4.71 × 10^−13^	12
Sample 5	1.776	6.50 × 10^−11^	32
Cabbage			
Control	0.414	ND	ND
Sample 1	0.392	ND	ND
Sample 2	0.474	ND	ND
Sample 3	1.051	9.46 × 10^−13^	8
Sample 4	0.427	ND	ND
Sample 5	0.719	4.22 × 10^−13^	14

Note: ND= not detected.

**Table 8:** Recoveries of *V. cholerae* cDNA spiked in Langat river water and cabbage samples with the voltammetric DNA biosensor via sandwich hybridization reaction (*n* = 4).

Sample	cDNA Concentration Added (µg/µL)	DNA Concentration Found (µg/µL)	Recovery (%)
River water			
Sample 2	-	1.20 × 10^−10^	-
Sample 2	9.21 × 10^−6^	9.35 × 10^−6^	101.56
Sample 4	-	4.71 × 10^−13^	-
Sample 4	9.21 × 10^−6^	8.98 × 10^−6^	97.17
Sample 5	-	6.50 × 10^−11^	-
Sample 5	9.21 × 10^−6^	9.06 × 10^−6^	98.34
Cabbage			
Sample 3	-	9.46 × 10^−13^	-
Sample 3	9.21 × 10^−6^	8.89 × 10^−6^	96.53
Sample 4	-	ND	-
Sample 4	9.21 × 10^−6^	9.16 × 10^−6^	99.45
Sample 5	-	4.22 × 10^−13^	-
Sample 5	9.21 × 10^−6^	9.08 × 10^−6^	98.59

Note: Recovery (%) = (A)/B × 100, where ‘A’ denotes the average DNA concentration determined by the DNA biosensor and ‘B’ denotes the actual DNA concentration added to the sample.

## Data Availability

Not applicable.
